# The N-terminal loop of IRAK-4 death domain regulates ordered assembly of the Myddosome signalling scaffold

**DOI:** 10.1038/srep37267

**Published:** 2016-11-23

**Authors:** Anthony C. G. Dossang, Precious G. Motshwene, Yang Yang, Martyn F. Symmons, Clare E. Bryant, Satty Borman, Julie George, Alexander N. R. Weber, Nicholas J. Gay

**Affiliations:** 1 Department of Biochemistry, University of Cambridge, Cambridge, CB2 1GA, UK; 2 Molecular Discovery Research, GlaxoSmithKline R&D, Stevenage, SG1 2NY, UK; 3 Department of Biochemistry, University of Pretoria, South Africa; 4 Department of Veterinary Medicine, University of Cambridge, Cambridge, CB2 1GA, UK; 5 Junior Research Group Toll-Like Receptors and Cancer, German Cancer Research Center (DKFZ), Im Neuenheimer Feld 580, 69120 Heidelberg, Germany; 6 Interfaculty Institute for Cell Biology, Department of Immunology, University of Tübingen, Auf der Morgenstelle 15, 72076 Tübingen, Germany

## Abstract

Activation of Toll-like receptors induces dimerization and the recruitment of the death domain (DD) adaptor protein MyD88 into an oligomeric post receptor complex termed the Myddosome. The Myddosome is a hub for inflammatory and oncogenic signaling and has a hierarchical arrangement with 6–8 MyD88 molecules assembling with exactly 4 of IRAK-4 and 4 of IRAK-2. Here we show that a conserved motif in IRAK-4 (Ser8-X-X-X-Arg12) is autophosphorylated and that the phosphorylated DD is unable to form Myddosomes. Furthermore a mutant DD with the phospho-mimetic residue Asp at this position is impaired in both signalling and Myddosome assembly. IRAK-4 Arg12 is also essential for Myddosome assembly and signalling and we propose that phosphorylated Ser8 induces the N-terminal loop to fold into an α-helix. This conformer is stabilised by an electrostatic interaction between phospho-Ser8 and Arg12 and would destabilise a critical interface between IRAK-4 and MyD88. Interestingly IRAK-2 does not conserve this motif and has an alternative interface in the Myddosome that requires Arg67, a residue conserved in paralogues, IRAK-1 and 3(M).

Pathogen associated molecular patterns (PAMPs) such as bacterial lipopolysaccharides and viral RNA bind to the Leucine Rich Repeat (LRR) extra cellular domains of the Toll-Like Receptors (TLRs) and initiate innate immune signalling^1^,^2^. The binding of PAMPs induces dimerization of the intracellular Toll/Interleukin-1 receptor (TIR) domains which provides a scaffold for the recruitment of downstream signal transducers^3^. With the exception of TLR3, the signalling adaptor protein MyD88 is recruited to initiate formation of the TLR post receptor complex.

MyD88 contains a N-terminal Death Domain (DD), a central linker region and a C-terminal TIR domain. It associates with activated receptors by TIR-TIR interactions and the DD of MyD88 then recruits the Interleukin-receptor associated kinases (IRAKs) by DD-DD interactions, to form a TLR post-receptor complex. Human cells have four IRAK paralogues with conserved N-terminal DD and C-terminal protein kinase. Recent studies showed that MyD88 is able to assemble a higher order DD complex of MyD88 and IRAK-4 DDs with stochiometries of 7:4 and 8:4 which was named the Myddosome^4^. Soon after this study, Lin *et al*.^5^ solved a crystal structure for a Myddosome variant. This revealed a hierarchical helical assembly of at least 6 MyD88, exactly 4 IRAK-4 and 4 IRAK-2 subunits arranged in four layers. Signal induced formation of Myddosome complexes leads to activation of an E3 ubiquitin ligase TRAF-6 and ultimately transcription factor NFκB, p38 kinase and ERK/MAP kinase (see ref. 1).

The structure of the Myddosome is a left-handed single helix with approximately 3.4 death domains per turn. Each DD has a minimum of two neighbouring DDs and a maximum of six adjacent DDs^5^,^6^. The interactions between the DDs can be classified into three types: Type I, II and III interactions^7^,^8^. The Myddosome complex is stabilised by type I and II interactions between the layers and type III interactions organise the structure into a helical assembly^5^.

Mutagenesis studies have identified specific residues that destabilise MyD88-IRAK interactions at each type of the interface^5^. Furthermore human polymorphisms in both MyD88 and IRAK-4 that cause life threatening pyogenic infections are defective in both Myddosome assembly and signalling through TLR and interleukin-1 pathways^9^,^10^. The MyD88 Ser34Tyr mutation causes steric interference at the type III IRAK-4/MyD88 interface and Arg98Cys disrupts the formation of a type II interface. Likewise a patient carrying an Arg12Cys mutation in the death domain of IRAK-4 has severely impaired innate responses including cytokine and ROS production^11^,^12^,^13^.

In unstimulated cells endogenous IRAK-4 is distributed in the cytosol and the protein kinase activity is inhibited. This is because activation depends on trans-autophosphorylation of the kinase activation loop at Thr345, a phospho-transfer event that requires the formation of an asymmetric kinase dimer. In solution this dimer has a K_d_ of 2.5 μM and thus does not occur to a significant extent at physiological levels of IRAK-4 expression. However when IRAK-4 forms into the Myddosome the kinase dimer is stabilised about 500 fold as a result of entropy effects caused by co-localization in the complex. Thus Myddosome assembly switches on the IRAK-4 kinase^14^.

The kinase activity of IRAK-4 plays a critical role in signalling mediated by the TLRs. Mice with an inactive IRAK-4 kinase are resistant to LPS induced shock and profoundly defective in cytokine and chemokine production in response to microbial stimuli of the TLRs. Activation of type 1 interferon by TLR7 and TLR9 is also impaired although there is a residual level of MyD88 dependent activation of NFκB. Conversely, MyD88-IRAK-4 signaling plays a critical role in B cell lymphomas when constitutively induced by MyD88 gain-of-function mutations which lead to constitutive Myddosome assembly^12^,^13^. These results show that the Myddosome induced activation of the IRAK-4 kinase is critical for innate immunity in the context of infection and oncogenesis^15^,^16^.

In this paper we report that the N-terminal loop of IRAK-4 plays a critical regulatory role in Myddosome assembly. We find that a sequence motif in this loop, which is not conserved in the other IRAK paralogues but is present in the *Drosophila* homologues tube and pelle, modulates the formation of a Type 2 interface with MyD88 that is required for Myddosome formation. We propose that phosphorylation of IRAK-4 Ser8 induces the N-terminal loop to form into an α-helix thereby preventing Arg12 from interacting with MyD88 Asp100.

## Results

### Characterisation and Kinetic analysis of full length IRAK-4

Full length IRAK-4 was expressed in insect cells and purified as described. The identity of the protein was confirmed by peptide mass fingerprinting with 20 peptide masses matched to 37% of IRAK-4 full length sequence. The exact mass of the protein was determined by intact LC-MS. The expected mass of IRAK-4 full length is 55250 ± 1 Da. The observed masses obtained by LC-MS were 55156 Da, 55235 Da, 55315 Da, 55395 Da and 55472 Da. These masses corresponded to an acetylated and des-Met form of IRAK-4 full length for the mass equal to 55156 Da and heterogeneous phosphoforms of IRAK-4 containing one to four phosphorylated residues with a mass addition of 79 ± 1 Da per phosphorylation.

### Isolation of singly phosphorylated Serine 8 IRAK-4 death domain

**Figure 1:**
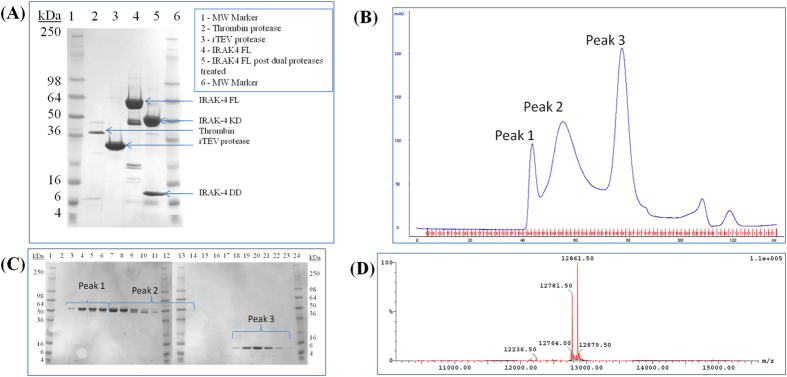
Purification of IRAK-4 death domain. (**A**) IRAK-4 full length was cleaved with thrombin and TEV proteases and analysed by XX% SDS-PAGE (**B**) Cleaved IRAK-4 was fractionated by gel filtration on 16/60 Superdex™ 75 pg (**C**) SDS-PAGE analysis of gel filtration peaks 1–3. (**D**) LC/MS spectrum showing deconvoluted masses of IRAK-4 DD purified in peak 3. The mass of 12782 Da corresponded to IRAK-4 death domain residues (−7) to 107 (see Methods). The mass of 12862 Da represents IRAK-4 death domain with a phosphorylated residue (+80 Da) (**C**).

Previous studies identified several phosphorylated residues in the IRAK-4 kinase and in addition a single site in an N-terminal peptide of the death domain^17^. We used LC-MS/MS analysis to unambiguously map this site as Ser8 in order to study the potential role of this phosphorylation in Myddosome assembly. A method was developed to separate the singly phosphorylated Ser8 IRAK-4 death domain. Treatment of full length IRAK-4 with thrombin protease revealed the cleavage of a small domain of approximately 12 kDa (Fig. 1A). After concentration of the samples to 15 mg.ml^−1^, the protease treated IRAK-4 was separated using a size exclusion column (Fig. 1B). The elution fractions corresponding to peaks 1, 2 and 3 were loaded and run on a 4–20% Tris-Glycine reducing SDS PAGE (Fig. 1C). Analysis of peak 3 by LC-MS revealed two species with M_r_ = 12782 ± 1 Da and 12862 ± 1 Da (Fig. 1D). The masses of the two species determined by LC-MS match to amino acids (−7) to 107 of IRAK-4 for the mass of 12782 Da and the addition of 80 Da corresponding to one phosphorylation adduct for the mass of 12862 Da. Amino acid sequence (−7) to 107 corresponds to the residual N-terminal amino acids Glu and Ala from the Tev cleavage site, a small linker (MetAspProGluPhe) between the Tev cleavage and the Met in position 1 and the N-terminal amino acids 1 to 107 of the death domain of IRAK-4. Thrombin was found to cleave IRAK-4 C-terminal to Lys107. The amino acid sequence AlaValProLys ‖ThrAla is not an obvious Thrombin cleavage site but this sequence can be recognised by Thrombin with a lower affinity^18^.

MALDI-MS/MS and LC-MS/MS analysis provided sequence data corresponding to 98% of the IRAK-4 DD and identified Serine 8 as the phosphorylated residue (supplementary information Tables 1–8). The stoichiometry of the phosphorylated versus non-phosphorylated species was calculated from the relevant ion intensities of corresponding ion chromatograms. The stoichiometry of the singly phosphorylated form was determined to be equal to 53%, with an error of ±10% (owing to the relative ionisation efficiency of each form).

**Figure 2:**
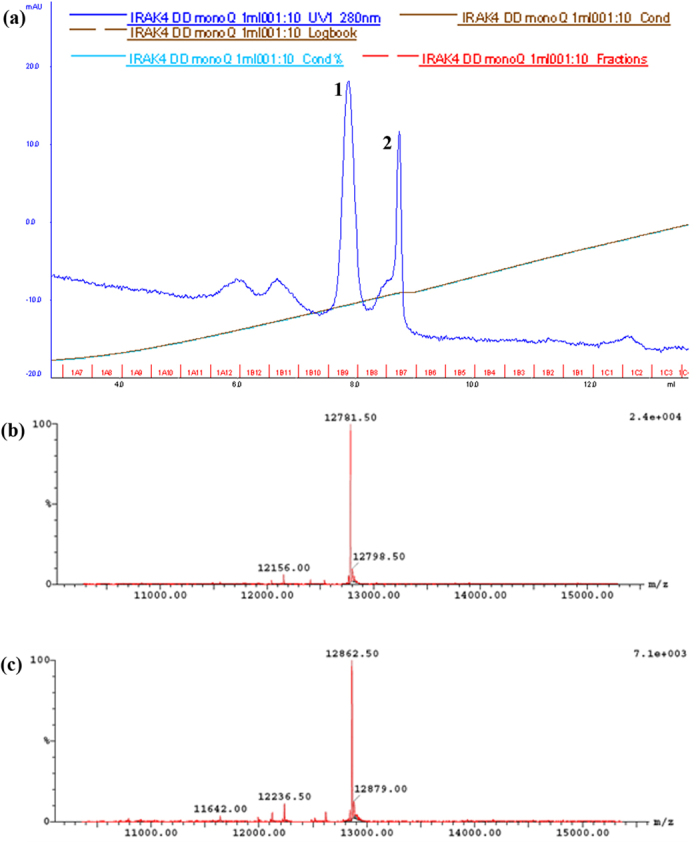
Separation of singly phosphorylated Ser8 IRAK-4 death domain. (**a**) Absorbance elution spectrum at 280 nm of IRAK-4 DD using a monoQ anion exchange column. Peaks 1 and 2 eluted at approximately 70 mM and 120 mM NaCl respectively. (**b**) LC/MS spectrum showing deconvoluted masses of IRAK-4 death domain corresponding to peak 1 fraction. The mass of 12782 Da is non-phosphorylated IRAK-4 death domain residues (−7) to 107. (**c**) LC/MS spectrum showing deconvoluted masses of IRAK-4 death domain corresponding to peak 2. The mass of 12863 Da corresponded to singly phosphorylated IRAK-4 death domain residues (−7) to 107.

Separation of singly phosphorylated and the non-phosphorylated IRAK-4 DD was achieved on a monoQ 5/50 anion exchange column. Proteins were eluted from the column with a linear gradient of 500 mM NaCl (Fig. 2a). Peaks 1 and 2 eluted at approximately 70 mM and 120 mM NaCl respectively. Fractions from each peaks were analysed by LC/MS. Peak 1 contained a single species with a mass of 12782 ± 1 Da corresponding to unphosphorylated IRAK-4 DD (Fig. 2b). Peak 2 was also a single species but with a mass of 12863 ± 1 Da (Fig. 2c) corresponding to a singly phosphorylated IRAK-4 DD, identified as located at Serine 8.

### IRAK-4 DD phosphorylated at serine 8 does not assemble into a Myddosome

**Figure 3:**
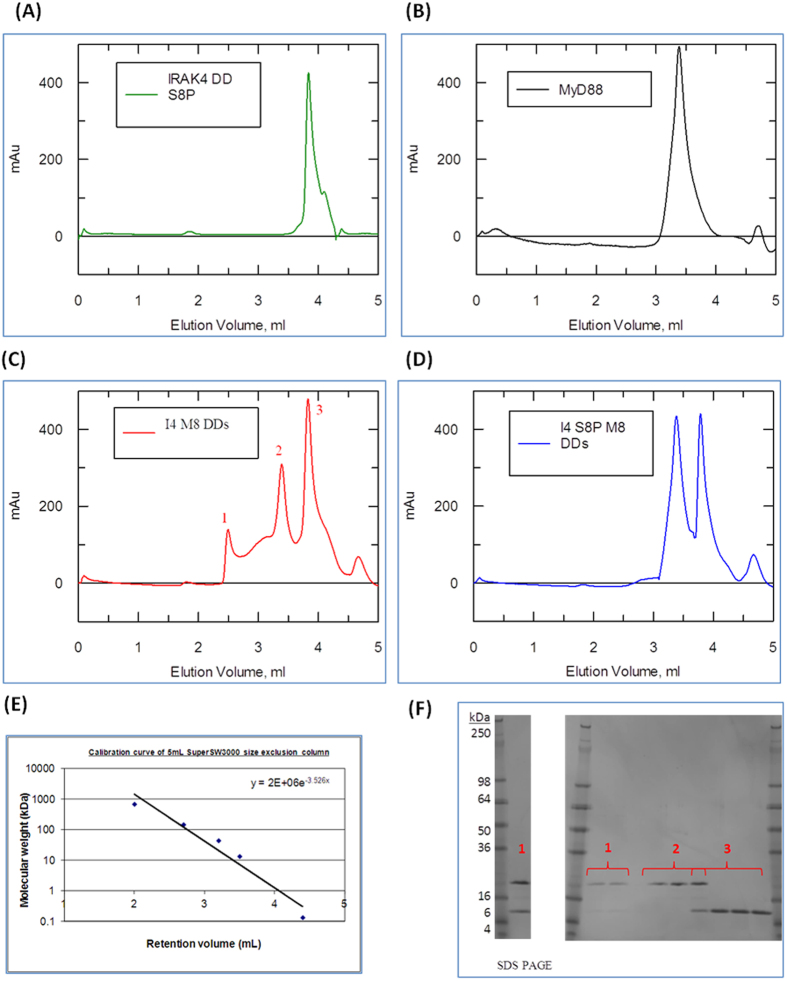
Phosphorylated Ser8 IRAK-4 death domain interferes with the Myddosome formation. (**A–D**) Absorbance Elution spectrum of a TSKgel SuperSW3000 analytical size exclusion column at 280 nm. (**A**) 0.5 mg.ml^−1^ of IRAK-4 death domain only. (**B**) 0.5 mg.ml^−1^ of MyD88 DD only. (**C**) 1:1 mix of MyD88 and non-phosphorylated IRAK-4 death domains concentrated to 0.5 mg.ml^−1^ prior to loading. (**D**) 1:1 mix of MyD88 and Ser8 phosphorylated IRAK-4 death domains concentrated to 0.5 mg.ml^−1^ prior loading. (**E**) Calibration of gel filtration using protein standard markers (**F**) 4–20% reducing Tris-Glycine SDS PAGE of fractions corresponding to peak 1, 2 and 3 of figure 15a. Fractions corresponding to peak 1 were concentrated using a 3 kDa MW cut-off Amicon Spin Filters for better visualisation of samples. One of three repeats is shown.

To study the role of Ser8 phosphorylation we carried out Myddosome assembly assays. Either unphosphorylated (Fig. 3C) or Ser8 phosphorylated (Fig. 3D) IRAK-4 DD was mixed with an equimolar amount of MyD88 DD. The samples were then concentrated to 0.5 mg.ml^−1^, loaded onto a calibrated 5 ml analytical size exclusion column and eluted at a flow rate of 0.3 ml.min^−1^.

Unphosphorylated IRAK-4 and MyD88 DD showed three peaks corresponding to approximate masses of 170 kDa, 35 kDa and 12 kDa (Fig. 3C). These fractions were run on reducing SDS-PAGE and revealed that peak 1 with an approximate mass of 170 kDa contained both IRAK-4 and MyD88 DDs, an assembled Myddosome. Peak 2 was equivalent to dimers of MyD88 DDs (Fig. 3B) and peak 3 to monomeric IRAK-4 DD (Fig. 3A). By contrast when the singly Ser8 phosphorylated IRAK-4 DD mixed with MyD88 DD only two peaks were observed which correspond to MyD88 DD dimers (35 kDa) and monomeric IRAK-4 DD (12 kDa) (Fig. 3D). These results showed that IRAK-4 death domain phosphorylated at Ser 8 is unable to assemble into a Myddosome.

**Figure 4:**
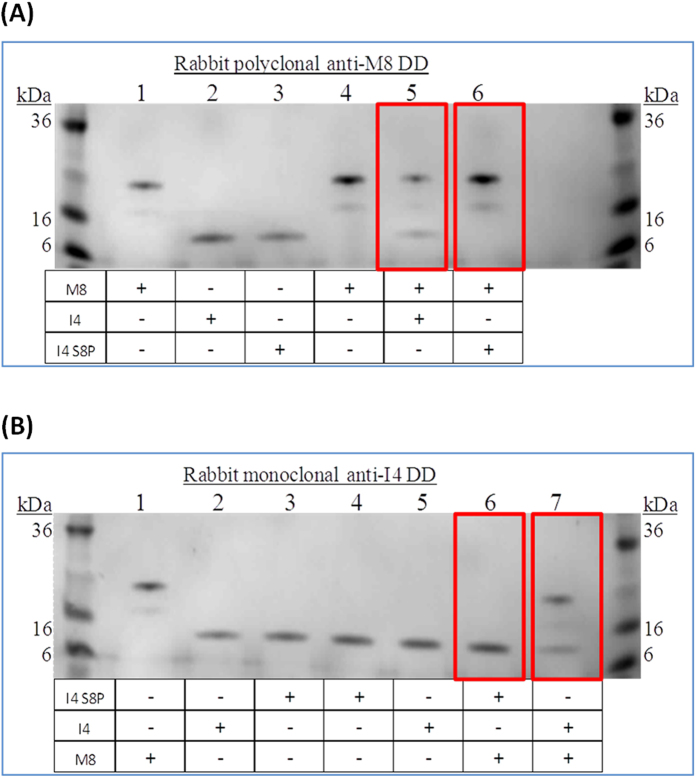
Reciprocal pull down assays confirm inability of Ser8-P death domain to interact with MyD88. (**A**) 4–20% reducing Tris-Glycine SDS PAGE of samples precipitated using a rabbit polyclonal anti-human MyD88 death domain antibody. Lane 1 to 3 are control lanes each loaded with 3 μg of the protein samples: lane 1-MyD88 DD, lane 2-non-phosphorylated IRAK-4 death domain, lane 3-Ser8 phosphorylated IRAK-4 death domain. Lanes 4 to 6 were loaded with the pull down experiment samples as indicated. (**B**) 4–20% reducing Tris-Glycine SDS PAGE of samples precipitated using a rabbit monoclonal anti-human IRAK-4 death domain antibody. Lane 1 to 3 were control lanes each loaded with 3 μg of the protein samples: lane 1 corresponded to MyD88 DD, lane 2-non-phosphorylated IRAK-4 death domain, lane 3-Ser8 phosphorylated IRAK-4 death domain. Lanes 4 to 7 were loaded with the pull down experiment samples as indicated. One repeat of three biological replicates is shown.

To confirm this conclusion, pull down experiments were carried out. MyD88 DD was incubated with either IRAK-4 DD or Ser 8 phosphorylated IRAK-4 DD at a final concentration of 0.5 mg.ml^−1^ overnight at room temperature. A rabbit polyclonal antibody specific for human MyD88 DD and goat anti-rabbit IgG magnetic beads were used to precipitate MyD88 protein complexes. After three washes the samples were analysed by SDS-PAGE to identify the complexes formed. The results show that only the non-phosphorylated IRAK-4 DD was able to bind MyD88 DD (Fig. 4A, lane 5). In reciprocal pull down experiments an antibody directed at the IRAK-4 N-terminal was used. This experiment confirms that only non-phosphorylated IRAK-4 DD was able to bind MyD88 DD (Fig. 4B, lane 7).

### A conserved sequence motif in IRAK-4 is a critical regulator of Myddosome assembly

**Figure 5:**
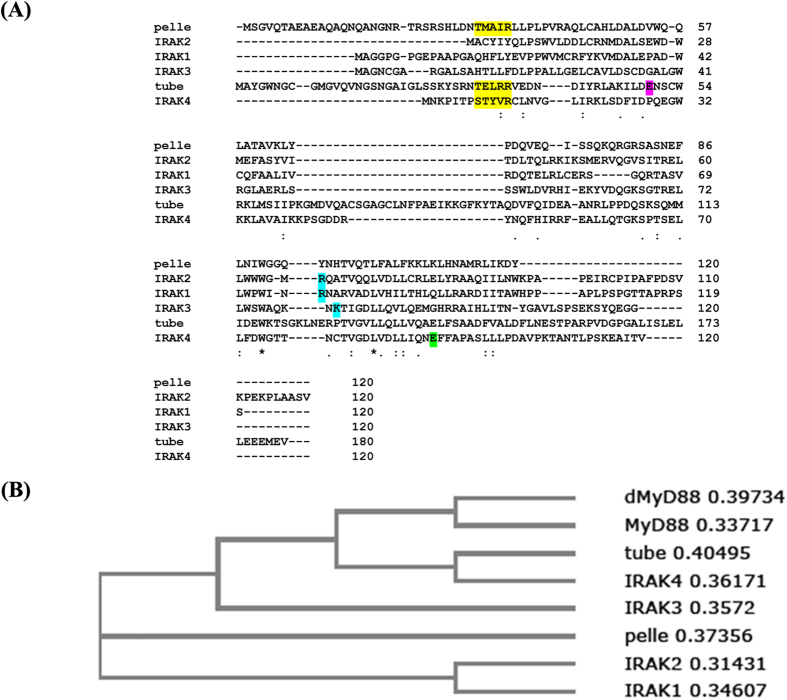
Specificity and regulation of Myddosome assembly mediated by Type II interfaces. (**A**) Sequence alignment of human MyD88 and IRAKs and *Drosophila* homologues dMyD88, tube and pelle. The conserved N-terminal loop motif is highlighted in yellow, tube Glu50 in purple, Arg67 of IRAK-2 in cyan and IRAK-4 Glu92 in green. (**B**) Phylogenetic relationships of DDs from Drosophila and human IRAK kinases and MyD88 adaptors.

**Figure 6:**
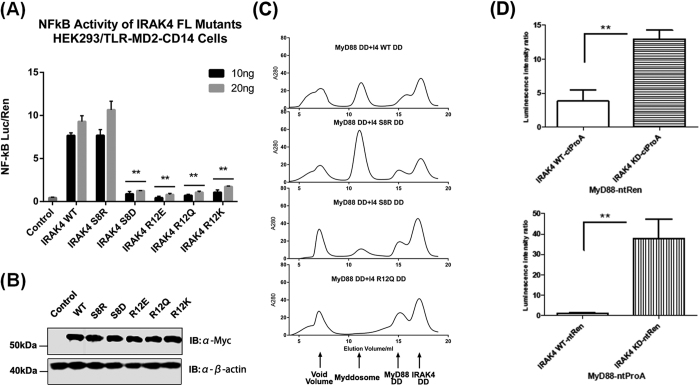
Regulatory function of the IRAK-4 N-terminal loop. (**A**) HEK293/TLR4-MD2-CD14 cells were transiently transfected with 10 ng or 20 ng of Myc-tagged IRAK4 constructs. Cells were harvested 24 hours post-transfection. NF-κB activation was quantified by dual luciferase assay. One representative of three independent experiments is shown. Statistical Analysis—p values were determined using multiple t test and designated with p < 0.01 (**) and p < 0.05 (*) One experiment of three biological replicates is shown. (**B**) Expression of all IRAK4 mutants is comparable with wild type IRAK4. HEK293/TLR4-MD2-CD14 cells were transfected with Myc-tagged IRAK4 constructs and analyzed by immunoblot (IB). One experiment of three biological replicates is shown. (**C**) Lumier assays: MyD88 and IRAK were tagged with renilla (Ren) or protein A (ProA) at the N- or C-terminal, as indicated. KD = kinase dead. One representative out of three identical experiments shown. **p < 0.01 measured by Student’s t-test.

**Figure 7:**
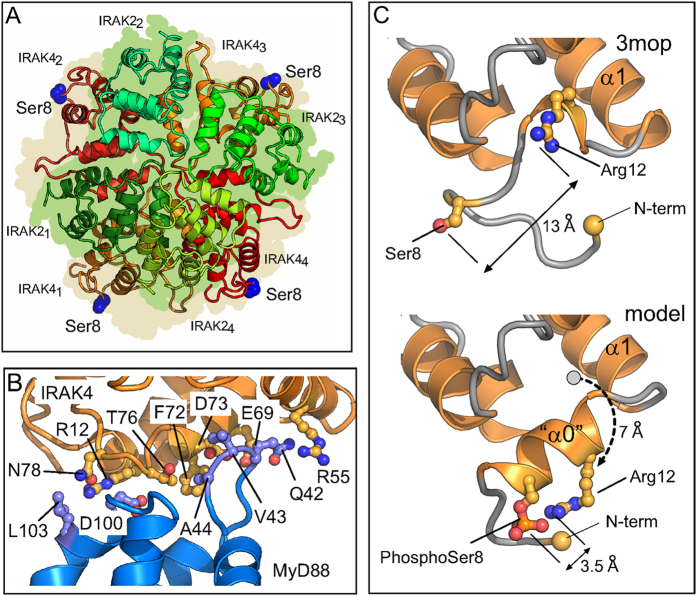
Specificity and regulation of Myddosome assembly mediated by Type II interfaces. (**A**) Cross section of the Myddosome structure (pdb:3mop) showing the four IRAK4 subunits (orange/red) and four IRAK 2 subunits (green/bluegreen). The peripheral location of IRAK4 Ser8 (blue atoms) is indicated. (**B**) Arg 12 forms part of a Type IIa DD-DD interface. The interaction between IRAK4 (g chain, pdb 3mop, orange ribbons and sidechains) and MyD88 (d chain, pdb 3mop, blue ribbons and sidechains). IRAK4 Arg12 and MyD88 Asp100 interact. (**C**) Modelled helix at N-terminus of IRAK4 stabilized by electrostatic interaction between phospho-serine 8 and arginine 12. Upper view shows N-terminus of pdb 3mop with 13 Å units between Arg12 and Ser8. Lower view shows modelled helix at N-terminus. Arg12 Calpha is moved 7 Å units from its position in pdb 3mop (dotted arrow). Arg12 sidechain can approach within 3.5 Å units of the modelled Phospho-Ser8 and is displaced from its interaction with MyD88 Asp100.

The Ser8 phosphorylation site characterised above lies on the outside surface of the Myddosome helix but does not contribute to a DD-DD interface. This suggests that the inability of Ser8P IRAK-4 to form a Myddosome is an indirect effect. We hypothesised that the phosphate group causes a conformational change that pulls this residue out from the Type 2 interfaces with MyD88 (Fig. 7B) causing a destabilisation of the complex. The Ser-X-X-X-Arg motif is highly conserved in vertebrate IRAK-4s but is not present in the other three IRAK paralogues (Fig. 5A,B). It is however found in the *Drosophila* IRAK homologues tube and pelle. To test this idea we carried out Myddosome assembly and signalling assays using several mutant IRAK-4 death domains (Fig. 6A–C). The phosphomimetic Ser8Asp mutation significantly impaired both assembly and signalling. By contrast the Ser8Arg mutation that introduces a positive charge enhanced the formation of Myddosomes *in vitro* and signalling to NFκB. A number of different mutants of Arg12 were also tested and all of these showed reduced levels of NFκB activation. One of these mutants, Arg12Gln did not support Myddosome assembly, consistent with the properties of the naturally occurring null mutation in IRAK-4 Arg12Cys (see below). We also tested the ability of kinase dead IRAK-4 to bind MyD88 with LUMIER protein-protein interaction assays (Fig. 6D) (see ref. 19). These experiments show that binding of the kinase dead IRAK-4 mutant to MyD88 is enhanced by about 5-fold as compared to the wild-type, consistent with the hypothesis that activation of IRAK-4 kinase regulates Myddosome assembly.

### Phosphorylation of serine 8 is predicted to induce formation of an α-helix

An analysis of the human IRAK-4 crystal structure reveals that Ser8 and Arg12 are 13 Å apart and thus unable to interact in this conformation (Fig. 7C). We therefore asked whether the N-terminal loop might adopt a different conformation. Indeed some IRAK-4 structures show residues 10–14 have a single turn of α- or 3/10 helix^20^ and phosphoserine residues stabilise α-helix formation especially when located at the N1 or 2 position^21^. Furthermore structural comparison shows that an additional helix is present at the N-terminus of homologous CARD domains (for example, human Caspase 9 CARD domain (pdb 4rhw^22^). In the light of this we modelled residues Pro7 to Leu14 as an α-helix, as described in ‘Methods’ (Fig. 7C). Structural validation of the model was carried out and is presented in Supplementary Information. This shows that all residues within the modeled segment adopt allowed dihedral angles and do not clash with other parts of the IRAK-4 DD structure, nor would the rearrangement create clashes with other subunits within the MyDDosome structure. Remarkably, analysis using FOLDX shows that the helical form is actually marginally more stable than the loop form, and that does not take account of the stabilization that will arise from the phosphoSer8-Arg12 electrostatic interaction. The structural rearrangement also moves the position of Arg12 by 7 Å and places it on the same side of the helix within 3.5 Å of phospho-Ser8. In this configuration Arg12 makes a highly favourable electrostatic bond with phospho-Ser8. This interaction is likely to be much more stable than the salt bridge that Arg12 makes with MyD88 Asp100 in the critical type 2 DD-DD interface in the Myddosome (Fig. 7B)^23^.

## Discussion

In recent years the discovery and characterisation of the Myddosome and other ‘supra molecular organising centres’^24^ such as the inflammasome^25^, the death inducing signalling complex (DISC)^26^ and the triffosome^1^ has lead to a new paradigm of inflammatory signal transduction. These highly oligomeric scaffolds assemble spontaneously in response to inflammatory stimuli such as pathogen associated molecular patterns. They provide a platform or hub that mediates complex patterns of signal transduction and cellular regulation. A key outstanding question is how pathway activation is coupled to scaffold assembly, a process that is hierarchical and intrinsically cooperative in nature.

In this study we show that the N-terminal loop in IRAK-4 plays a critical role in the sequential assembly of the Myddosome and that autophosphorylation may regulate the assembly process. The residue Arg12 is mutated to cysteine in a French patient who is trans-heterozygous with an IRAK-4 frameshift allele^11^. He has a history of severe infections by pyogenic bacteria and is completely defective in MyD88 dependent cytokine responses. In the Myddosome structure Arg12 makes a major contribution to the IRAK-4–MyD88 Type II interfaces, binding to the MyD88 residues Asp100 and Leu103 (Fig. 7B). A recent report also shows that the Arg12Cys mutant cannot signal to NFκB or assemble into a Myddosome^27^. The critical importance of Arg12 for signalling is confirmed by our finding that substitution with a negatively charged residue, an amide or even positively charged lysine profoundly inhibits the ability of IRAK-4 to activate NFκB in a constitutive signalling assay. The Arg12Gln mutant is also unable to assemble into Myddosomes.

We also find that another residue in the N-terminal loop of IRAK-4 regulates MyD88 dependent signalling. We have identified Serine 8 as a target for autophosphoryation by IRAK-4 kinase and purified the death domain containing phospho-serine8. Remarkably the phosphorylated death domain is unable to assemble Myddosomes. Furthermore the phospho-mimetic Ser8Asp mutant is strongly impaired in signalling assays and Myddosome assembly whereas a mutation that introduces a positive charge, Ser8Arg, is unaffected in signalling and forms Myddosomes *in vitro* with enhanced kinetics. Serine 8 does not participate in DD-DD interactions in the Myddosome directly although it is interesting to note its proximity to Arg12. An attractive explanation for the regulatory properties of Serine 8 reported here is that the phosphate group or a negatively charged amino acid can induce a conformational change that pulls Arg12 out from the interfaces with MyD88 causing a destabilisation of the complex. Our modelling study strongly predicts that phosphorylation of Ser8 will cause residues 2–14 to adopt an α-helical configuration stabilised by a salt bridge with Arg12. In future work we plan to confirm this by solving the crystal structure of the IRAK-4 phospho death domain.

This general scheme of phosphorylation induced cellular regulation is seen in other contexts. For example the enzyme for glycogen breakdown, phosphorylase, is activated by phosphorylation of Ser14 which then forms electrostatic interactions with two arginine residues^28^. This leads to a conformational rearrangement that causes a loop blocking the active site to fold into an α-helix allowing access to substrate.

The N-terminal loop motif of IRAK-4 is not conserved in the three human paralogues, IRAK-1, IRAK-2 and IRAK-3(M) (Fig. 5D). In the Myddosome structure, the equivalent Type 2 interaction between the IRAK-4 and IRAK-2 layers is mediated by Glu92 and Arg67 respectively. Of note Arg67 is at the C-terminus of helix 4 spatially quite close to the IRAK-2 N-terminal loop and this residue is not conserved in IRAK-4 (Fig. 5A). Mutation of Arg67 to Asp completely abolishes the binding of IRAK-2 to IRAK-4 (see (Fig. S9 in ref 5). Therefore this residue is essential for formation of a Type 2 interface that is structurally similar but not identical to that formed between IRAK-4 Arg12 and MyD88 Asp100. These features are likely to confer specificity for the sequential assembly of the MyD88, IRAK-4 and IRAK-2 subunits that occurs during assembly of the Myddosome. A phylogenetic tree shows that IRAK-1 and 2 are more closely related to each other than to IRAK3 and 4 and it is interesting that the residues contributing to the IRAK-2 Type IIa interface are conserved in IRAK1 (Fig. 5A). Likewise IRAK-3(M) lacks the N-terminal IRAK-4 Ser/Thr-X-X-X-Arg motif and has a positively charged amino acid (Lys79) equivalent to IRAK-2 Arg67 that could form an electrostatic interaction in a Type 2 interface with IRAK-4. Taken together these observations suggest that IRAK-2, IRAK-1 and IRAK-3 may assemble into the third layer of the Myddosome interchangeably. It is also possible that IRAK-3(M) may be able to form a fourth layer in an IRAK-2 Myddosome as the critical Type IIb residue is conserved in IRAK-2 (Glu82), consistent with a previous proposal by Zhou *et al*.^29^.

As shown in Fig. 5A, both tube adaptor and pelle kinase retain the Ser/Thr-X-X-X-Arg motif. Phylogenetic analysis shows that tube is most closely related to IRAK-4 but has lost the kinase domain and pelle is evolutionarily related to IRAK-1/2 (Fig. 5B and ref. 30). Activation of the *Drosophila* Toll pathway leads to formation of a simpler heterotrimeric complex of dMyD88 with adaptor tube and pelle that is topologically equivalent to a segment of the Myddosome structure (one subunit of MyD88, IRAK-4 and IRAK-2 arranged end to end; see refs 31 and 5). Tube Arg34 interacts with dMyD88 Asp166 to form a Type 2 interface that is equivalent to that between IRAK-4 Arg12 and MyD88 Asp100 (Fig. 7B, ref. 5). Both of these residues are essential for the formation of a stable tube-dMyD88 complex and for signalling function^32^. Similarly the pelle residue Arg35, which is Tyr in IRAK-2 (see Fig. 5A) interacts with tube Glu50 in a type II DD-DD interface^33^. Neither the Pelle mutant Arg35Glu nor the tube mutant Glu50Lys is active for dorsoventral patterning in *Drosophila* embryos but strikingly the two charge reversal mutations expressed together (pelle Arg35Glu; tube Glu50Lys) are capable of restoring high and possibly unregulated levels of Toll signalling activity to produce the most ventral elements of the embryo. Interestingly another essential residue for formation of the Type II dimer is Glu140, equivalent to Glu82 and Glu92 in IRAK-2 and 4 (see above)^33^. It should also be noted that in yeast 2-hybrid assays kinase inactive pelle interacts 30x more strongly with tube than the active form, a finding that is consistent with phosphorylation induced downregulation^34^.

In conclusion these studies provide insight into the hierarchical process of Myddosome assembly and how this supra-molecular organising centre has evolved from the simpler linear structures found in invertebrates. It is interesting to note that in the *Drosophila* Toll pathway the pelle kinase acts directly on IκB homologue cactus^35^. The evolution of the Myddosome has allowed regulation of the Toll signalling pathway to diversify, perhaps reflecting the need for complex control of potentially damaging inflammatory responses in long living vertebrate organisms.

## Materials and Methods

### Cloning

Purified recombinant His-TEV-IRAK-4 was previously described (18). Briefly, cDNA for full length IRAK-4 was PCR amplified with primers such as to create an EcoRI site at the 5′ terminus, and an XbaI site at the 3′ terminus. The PCR product, and the vector pFastBacHTa, were restriction digested with EcoRI and XbaI enzymes. The digested vector was alkaline phosphatase treated and the digested PCR product was ligated into the multiple cloning site. The cDNA vector containing the IRAK-4 gene sequence was transformed into DH10Bac *E.coli* cells. Fresh transformants were used to inoculate cultures from which Bacmid DNA was isolated.

A cDNA of MyD88 was purchased from Invitrogen (clone 3900351) in a Gateway^®^ compatible vector. PCR was performed to create DNA fragments representing MyD88 containing the death domain and intermediate domain (aa 1 to 158). The PCR products were inserted into a pENTR^TM^ vector before recombination into a pDEST^TM^ vectors carrying a 6xHis-GST tag and a Tev cleavage site on the N-terminus.

### Cell culture and viruses

Transfections of *Spodoptera frugiperda* (Sf9) cells with the Bacmid DNA was performed using Invitrogen Cellfectin II reagent following the manufacturer recommendations. All Sf9 cell culture was performed in EX-CELL 420 media (SIGMA-ALDRICH # 14420 C) and in the absence of FBS. P0 virus was harvested and amplified to give P1 high titre virus. The P1 virus was used to infect Sf9 insect cells at a density of 3 × 10^6^ cells.ml^−1^ with a multiplicity of infection (MOI) of 3 virus particles per cell. Infected cells were incubated for 48 hours at a temperature of 27 °C and a rotation of 120 rpm in a total volume of 2 L in 3 L maximum capacity flasks. Infected cells were assessed during the incubation time for both their viability level and cell diameter with regards to infection levels.

Recombinant 6xHis-GST-Tev-MyD88 DD was successfully expressed in BL21 Star™ (DE3) *E.coli* cells (Invitrogen # C6010–03) using Turbo Broth media (Molecular Dimensions # MD12-104-1) with the addition of 1% glucose and 100 μg.ml^−1^ Ampicillin, incubated at 37 °C for 4 hours at 200 rpm followed by the addition of 500 μM IPTG before incubation at 18 °C overnight.

### Protein purification

Cells pellets expressing full length IRAK-4 were suspended in buffer A containing 20 mM Tris pH 8.0, 300 mM NaCl, 1 mM TCEP, 10% glycerol, 1 mM Benzamidine, 0.2 mM PMSF and Calbiochem Complete protease inhibitor III (1 ml.L^−1^). The cells were then lysed by dounce homogenisation using 20 strokes per 50 ml on ice. The suspension was then centrifuged at 100,000 g for 90 min. Two 5 ml HisTrap^®^ HP columns, connected in series were pre-equilibrated with 10 column volumes (CV) of buffer A before loading the soluble IRAK-4 supernatant at 1 ml.min^−1^. The column was then washed with 10 CV of buffer B (buffer A + 1 M NaCl), followed by 10 CV of buffer C (buffer A + 40 mM Imidazole). The column was then eluted at 2 ml.min^−1^ over 10 CV collecting 5 ml fractions with buffer D (buffer A + 300 mM Imidazole). All fractions of interest were analysed using reducing SDS-PAGE. Fractions were then pooled and concentrated using an Amicon Spin Filter with 30 kDa MW cut-off to a 15 ml final volume. The concentrated sample containing IRAK-4 was run on a HiLoad 16/60 Superdex 200 prep grade size exclusion column, pre-equilibrated with 1.2 CV buffer containing 50 mM Tris pH 7.4, 150 mM NaCl, 1 mM TCEP and 0.1 mM EDTA at 2 ml.min^−1^. The column was then loaded and eluted at 2 ml.min^−1^ over 1.2 CV collecting 2 ml fractions. The fractions of interest were. Fractions of interest were pooled and concentrated using Amicon spin filters with a 30 kDa MW cut-off to a concentration of 1.1 mg.ml^−1^.

Cell pellets expressing MyD88 DD were resuspended in buffer containing 50 mM Tris-HCl pH 8, 150 mM NaCl, 10% glycerol, 1 mM DTT and protease inhibitor cocktail. The cells were then lysed by ultrasonic treatment and centrifuged for 90 minutes at 100,000 g. The soluble fraction was then bound to a pre-equilibrated 5 ml GSTrap HP^TM^ column and incubated overnight with 6xHis-TeV protease for on-column tag cleavage. A pre-equilibrated 5 ml HisTrap HP^TM^ column was then added in series with the GSTrap column to elute the cleaved protein and retain both the 6xHis tag and the 6xHis-TEV protease. The eluted MyD88 DD fractions were pooled and loaded into a pre-equilibrated 16/60 Superdex™ 75 pg size exclusion column to separate remaining contaminant protein and determine the approximate oligomeric state of MyD88 DD.

### Proteolysis and purification of phosphorylated Serine 8 IRAK-4 DD

Full length IRAK-4 at 1.1 mg.ml^−1^ was treated with 120 units.ml^−1^ of Thrombin and 3 uM of rTeV incubated overnight at 4 °C. After concentration of the samples using an Amicon Ultra 3 kDa MW cut-off filter from Millipore to 1.5 mg.ml^−1^, the protease treated IRAK-4 samples were separated using a 16/60 Superdex™ 75 pg size exclusion column. The elution fractions corresponding to IRAK-4 DD, identified on a 4–20% Tris-Glycine reducing SDS PAGE, were pooled together, concentrated to 1 mg.ml^−1^ using an Amicon Ultra 3 kDa MW cut-off filter from Millipore.

Separation of the singly phosphorylated and the non-phosphorylated Ser8 IRAK-4 DD was achieved by loading the heterogeneously phosphorylated fractions (previously buffer exchanged in 50 mM Tris-HCL pH 7.4, 1 mM DTT and 0.1 mM EDTA) on a pre-equilibrated monoQ 5/50 anion exchange column. Elution from the column was achieved with a linear gradient of 500 mM over 15 CV.

### NFκB luciferase reporter and lumier assays

These assays were carried out as previously described^19^. In brief the LUMIER method relies on an affinity purification of Protein A tagged proteins via magnetic IgG-beads (Dynabeads M-280, sheep anti-rabbit IgG, Invitrogen). Co-purified interacting proteins were detected via Renilla luciferase activity.

The IRAK-4 kinase dead mutant (KK213AA) was described^36^.

### Liquid Chromatography–Mass Spectometry (LC-MS)

Protein intact masses were derived using LC-MS on a Waters 2795 LCT Premier using ESI. Detection limits were set at 800–2500 m/z. The column used was a 2.1 × 150 mm PLRP-S 8 μm 1000 A (Polymer Labs) with a column temperature of 60 °C being maintained during the sample run. Sample loading was 50 pmoles and a flow rate of 0.25 ml.min^−1^ was used. Buffer A was constituted of 5% Acetonitrile (far UV) and 0.05% TFA, whilst buffer B was constituted of 95% acetonitrile (far UV) and 0.05% TFA. A gradient was run as follows; start 95% A, 5% B; 25 minutes 15% A, 85% B; 30 minutes 10% A, 90% B; 32 minutes 5% A, 95% B and 35 minutes 95% A, 5% B.

### SDS-PAGE

The protein was electrophoresed on SDS-PAGE 4–12% Tris NuPage gel under reducing conditions using MES running buffer and stained with SimplyBlue™ SafeStain (InVitrogen) unless otherwise stated.

### Sequencing of IRAK-4 death domain by MALDI-TOF MS and ESI MS-MS

Protein bands corresponding to IRAK-4 DD were excised from the polyacrylamide gel and reduced with DTT, before the cysteine residues were carboxyamidated and digested *in situ* with trypsin, chymotrypsin and endoproteinase Asp-N according to a modification of the method of Shevchenko *et al*.^37^,^38^.

### Preparation of Sample for MALDI-TOF MS Analysis

A 1 μl sample of the liquid surrounding the gel pieces was mixed with 1 μl of a matrix solution containing 60% (v/v) acetonitrile, 0.5% (v/v) trifluoroacetic acid and 6 mg.ml^−1^ α-cyano-4-hydroxycinnamic acid in the bottom of a microfuge tube. Immediately after, 1 μl of the mixture was spotted onto a 384-well stainless steel MALDI-TOF mass spectrometer target and allowed to dry, leaving a crystallized mixture of sample and matrix on the target for analysis. A peptide calibration standard solution (Bruker Daltonics, Bremen, Germany) was spotted in a similar manner adjacent to the samples.

### LC-ESI Mass Spectrometry

Five microliters of a digested sample was diluted with 5 μl of 0.1% formic acid. Three microliters was injected onto an Agilent HPLC coupled to a Bruker Amazon ion-trap mass spectrometer. The sample was separated on a reversed-phase column (Imtakt 3 μm Cadenza CD-C18, 150 mm × 0.3 mm, # CD0F5) using a 1 hour elution gradient from 2% acetonitrile, 0.1% formic acid to 60% acetonitrile, 0.1% formic acid. Automatic ms and ms/ms peak selection was used, and the data collected between 2 to 45 min were used to generate compound lists for database searching. Blank solutions containing 0.1% formic acid, 50% acetonitrile were injected between each run to elute any residual bound material from the previous sample off the column. The detector and mass calibration were carried out by injecting 0.5 ml tune mix, diluted 1:50 with acetonitrile, by means of a syringe pump. Additional analysis was performed on the Orbitrap mass spectrometer using an Acclaim PepMap RSLC 50 μm × 15 cm, C18, 2 μm, 100 A column.

### Pull down experiments

These experiments used magnetic beads conjugated to appropriate antibodies that can be separated and washed using a magnetic rack. 100 μl of MagnaBind™ Goat Anti-Rabbit IgG from Piercenet (# 21356) were used and washed 3 times in 0.5 ml of buffer containing 20 mM Tris-HCl, pH 7.4, 50 mM NaCl, 1 mM DTT and 0.1 mM EDTA in a 0.5 ml microcentrifuge tube. 100 μl of a solution containing 0.5 to 1 mg.ml^−1^ of the rabbit antibody raised against the protein of interest was added to beads and incubated at +4 °C for a minimum of 1 hour. The beads were washed 3 times in 0.5 ml buffer before 200 μl of samples containing the protein complexes were added to the beads and incubated at +4 °C for a minimum of 2 hours on a rotating mixer at low speed (<20 rpm) and washed 4 times with 0.5 ml of buffer solution. The bead-Antibody-Antigen complex was then mixed in 50 μl SDS loading buffer solution.

### Kinase activity assay

Kinase activity was monitored by the phosphorylation of the peptide substrate 5-TAMRA (5-carboxytetramethylrhodamine)-ERMRPRKRQGSVRRRV-CONH2 (Molecular Devices # R7329) using an IMAP™ fluorescence polarisation based assay (21). The reactions were performed in a 384 well black Greiner low volume plate (cat # 784201) at 25 °C in 50 mM Tris-HCL buffer (pH 7.4), 50 mM NaCl, 1 mM DTT and 1 mM CHAPS. *K*_*m app, ATP*_ was determined by preparing solutions of ATP 2-fold the final assay concentrations plus a buffer blank in reaction buffer. Five microliters of the ATP solutions were added to a 384 well black Greiner plate. A solution containing 200 nM peptide substrate and 5 nM IRAK-4 was prepared and 5 μl was added to all wells to initiate the reaction. Several replicates were made in order to quench the reaction at each time points by the addition of 10 μl stop solution containing 1 x Progressive Binding solution (containing IMAP™ beads) prepared in 100% binding buffer A. The plate was then read after 1 hour incubation on a 384 LJL Acquest from Molecular Devices under a TAMRA fluorescence polarisation protocol (excitation = 540 nm, emission = 575 nm). Fluorescent polarisation data was then plotted as a function of time to determine linearity at each ATP concentration tested.

### Sequence alignments and phylogenetic trees

These were generated using Clustal Omega (http://www.ebi.ac.uk/Tools/msa/clustalo/).

### Modelling methods

The pdb 3mop IRAK4 G chain residues 2–15 were re-built as a helix using UCSF Chimera 1.9. PhosphoSer atom positions and sidechain rotamers were chosen from the Coot library and regularized^39^.

## Additional Information

**How to cite this article**: Dossang, A. C. G. *et al*. The N-terminal loop of IRAK-4 death domain regulates ordered assembly of the Myddosome signalling scaffold. *Sci. Rep.* **6**, 37267; doi: 10.1038/srep37267 (2016).

**Publisher's note:** Springer Nature remains neutral with regard to jurisdictional claims in published maps and institutional affiliations.

## Supplementary Material

Supplementary Information
