# Supplementary material for: The N-terminal loop of IRAK-4 death domain regulates ordered assembly of the Myddosome signalling scaffold
Source: Sci Rep. 2016 Nov 23;6:37267. doi: 10.1038/srep37267 (PMC5120336; doi:10.1038/srep37267)
Supplement: Supplementary Information [file srep37267-s1.pdf]

The N-terminal loop of IRAK-4 death domain regulates ordered assembly of the Myddosome signalling scaffold

Anthony C.G. Dossang[*†1], Precious G. Motshwene[*‡1], Yang Yang*[1], Martyn F. Symmons*, Clare E. Bryant[§], Satty Borman[†], Julie George[||], Alexander N. R. Weber[¶] and Nicholas J. Gay*[2]

SUPPLEMENTARY METHODS - PHOSPHOSITE MAPPING.

Phosphosite mapping analysis was then carried out on the isolated IRAK-4 death domain. The band corresponding to IRAK-4 DD extracted from the reducing SDS-PAGE gel was digested using Trypsin, Chymotrypsin and AspN endoproteinases. The analysis was carried out using MALDI-TOF MS on an Ultraflex mass spectrometer and by LC-ESI MS on an Agilent HPLC coupled to a Bruker Amazon ion-trap and an Orbitrap mass spectrophotometer where appropriate. One hundred and twenty nine peptides were identified by LC/MS-MS and 44 peptides were identified by MALDI-TOF MS covering 100 % of the IRAK-4 DD (-7) to 107 (Supplementary data tables 1 to 6). The stoichiometry of both species was calculated from the relevant ion intensities of corresponding ion chromatograms. The stoichiometry of the singly phosphorylated form was determined to be equal at 53 % with an error as high as ± 10 % (as it depends on the relative ionisation efficiency of each form; phosphorylated and non-phosphorylated). In total 16 peptide string masses identified confirmed single phosphorylation of Serine 8 in IRAK-4 DD (Supplementary data tables 1 to 6). Phosphorylation at Serine 8 was further confirmed by the fragmentation of the peptide GAMDPEFMNKPITPSTYVR by MS/MS as shown in supplementary tables 7 and 8.

Table 1: a) List 1 of 2 of peptides identified by LC / MS – MS after digestion of IRAK-4 DD using Chymotrypsin. b) List 2 of 2 of peptides identified by LC / MS – MS after digestion of IRAK-4 DD (Section 3.2.4) using Chymotrypsin.

(a)

Acc. #	Protein ID	Pep. #	% Cov.	Score
9947	IRAK4 DEATH DOMAIN	57	94%	*1182*

GAMDPEFMNK PITPSTYVRC LNVGLIRKLS DFIDPQEGWK KLAVAIKKPS
GDDRYNQFHI RRFEALLQTG KSPTSELLFD WGTTNCTVGD LVDLLIQNEF
FAPASLLLPD AVPK

Red = MS-MS **Underline = PMF**

Comments: Individual ions scores > 23 indicate identity or extensive homology (p<0.05).

Score	Peptide	Modifications	Obs. m/z	Delta	MS-MS?	PMF?
66	-.GAMDPEFMNKPITPSTYVRCL.N	Phospho (Y); 2 Oxidation (M)	847.04	0.0022	☒	☐
40	-.GAMDPEFMNKPITPSTY.V	2 Oxidation (M)	965.93	0.0031	☒	☐
33	-.GAMDPEFMNKPITPSTYVRCLNVGL.I	Phospho (ST); 2 Oxidation (M)	974.78	0.0029	☒	☐
(23)	-.GAMDPEFMNKPITPSTY.V	Oxidation (M)	957.94	0.0031	☒	☐
(30)	-.GAMDPEFMNKPITPSTY.V	2 Oxidation (M)	965.93	0.0000	☒	☐
(30)	-.GAMDPEFMNKPITPSTYVRCL.N	Phospho (ST); Oxidation (M)	1,262.06	0.0040	☒	☐
(32)	-.GAMDPEFMNKPITPSTY.V	Phospho (Y); 2 Oxidation (M)	1,005.92	0.0006	☒	☐
(34)	-.GAMDPEFMNKPITPSTY.V	Oxidation (M)	957.93	-0.0002	☒	☐
(35)	-.GAMDPEFMNKPITPSTY.V		949.94	0.0001	☒	☐
(49)	-.GAMDPEFMNKPITPSTYVRCL.N	Phospho (ST); Oxidation (M)	841.71	0.0019	☒	☐
(56)	-.GAMDPEFMNKPITPSTYVRCL.N	Phospho (ST)	836.38	0.0029	☒	☐
56	F.MNKPITPSTY.V	Oxidation (M)	584.29	0.0002	☒	☐
(32)	F.MNKPITPSTY.V	Phospho (ST); Oxidation (M)	624.27	0.0012	☒	☐
(50)	F.MNKPITPSTY.V		576.29	0.0030	☒	☐
42	Y.VRCLNVGL.I		465.76	-0.0006	☒	☐
46	L.NVGLIRKLSDFIDPQEGW.K		696.37	0.0009	☒	☐
43	L.NVGLIRKLSDF.I		631.37	0.0004	☒	☐
30	L.NVGLIRKL.S		456.80	-0.0005	☒	☐
(30)	L.NVGLIRKLSDFIDPQEGW.K		696.37	0.0016	☒	☐
(32)	L.NVGLIRKLSDFIDPQEGW.K		696.37	0.0000	☒	☐
(34)	L.NVGLIRKLSDFIDPQEGW.K		696.37	-0.0011	☒	☐
34	L.IRKLSDFIDPQEGW.K		568.63	-0.0002	☒	☐
28	L.IRKLSDFIDPQEGWKKL.A		519.04	-0.0017	☒	☐
(23)	L.IRKLSDFIDPQEGW.K		852.44	-0.0006	☒	☐
35	L.SDFIDPQEGWKKL.A		781.90	0.0000	☒	☐
31	L.SDFIDPQEGW.K		597.26	-0.0010	☒	☐
(27)	L.SDFIDPQEGWKKL.A		521.60	-0.0012	☒	☐
33	W.KKLAVAIKKPSGDDRY.N		448.01	-0.0005	☒	☐
(26)	W.KKLAVAIKKPSGDDRY.N		448.01	0.0001	☒	☐
67	L.AVAIKKPSGDDRY.N		710.38	0.0005	☒	☐
37	L.AVAIKKPSGDDRYNQF.H		904.97	0.0054	☒	☐

(b)

Acc. #	Protein ID	Pep. #	% Cov.	Score
9947	IRAK4 DEATH DOMAIN	57	94%	*1182*

GAMDPEFMNK PITPSTYVRC LNVGLIRKLS DFIDPQEGWK KLAVAIKKPS
GDDRYNQFHI RRFEALLQTG KSPTSELLFD WGTTNCTVGD LVDLLIQNEF
FAPASLLLPD AVPK

Red = MS-MS **Underline = PMF**

Comments: Individual ions scores > 23 indicate identity or extensive homology (p<0.05).

Score	Peptide	Modifications	Obs. m/z	Delta	MS-MS?	PMF?
37	L.AVAIKKPSGDDRYNQF.H		904.97	0.0054	☒	☐
(24)	L.AVAIKKPSGDDRYNQF.H		603.65	0.0035	☒	☐
(24)	L.AVAIKKPSGDDRYNQF.H	Phospho (ST)	630.30	0.0002	☒	☐
(31)	L.AVAIKKPSGDDRY.N		473.92	-0.0008	☒	☐
48	F.EALLQTGKSPTSELL.F		793.94	0.0005	☒	☐
43	F.EALLQTGKSPTSEL.L		737.39	-0.0007	☒	☐
(42)	F.EALLQTGKSPTSEL.L		737.39	0.0012	☒	☐
47	L.LQTGKSPTSELLF.D		710.89	0.0001	☒	☐
24	L.LQTGKSPTSEL.L		580.81	0.0006	☒	☐
36	L.QTGKSPTSEL.L		524.27	-0.0004	☒	☐
34	L.QTGKSPTSELLF.D		654.35	0.0001	☒	☐
29	L.QTGKSPTSELL.F		580.81	0.0003	☒	☐
30	W.GTTNCTVGDLVDLLIQNEF.F		1,055.01	0.0029	☒	☐
41	L.LIQNEFFAPASL.L		675.36	0.0002	☒	☐
27	L.IQNEFFAPASLL.L		675.36	0.0007	☒	☐
25	L.IQNEFFAPASL.L		618.82	0.0001	☒	☐
71	F.FAPASLLLPDAVPK.-		719.92	0.0007	☒	☐
(25)	F.FAPASLLLPDAVPK.-		719.92	0.0005	☒	☐
(27)	F.FAPASLLLPDAVPK.-		719.92	0.0008	☒	☐
(29)	F.FAPASLLLPDAVPK.-		719.92	0.0001	☒	☐
(33)	F.FAPASLLLPDAVPK.-		719.92	0.0012	☒	☐
(38)	F.FAPASLLLPDAVPK.-		719.92	0.0006	☒	☐
(54)	F.FAPASLLLPDAVPK.-		719.92	0.0011	☒	☐
(60)	F.FAPASLLLPDAVPK.-		719.92	0.0014	☒	☐
(66)	F.FAPASLLLPDAVPK.-		719.92	-0.0003	☒	☐
57	F.APASLLLPDAVPK.-		646.38	-0.0003	☒	☐
49	L.LPDAVPK.-		370.22	0.0001	☒	☐

Table 2: a) List 1 of 2 of peptides identified by LC / MS – MS after digestion of IRAK-4 using Trypsin. b) Part 2 of 2 of peptides identified by LC / MS – MS after digestion of IRAK-4 DD using Trypsin.

(a)

Acc. #	Protein ID	Pep. #	% Cov.	Score
9947	IRAK4 DEATH DOMAIN	59	62%	*645*

GAMDPEFMNK PITPSTYVRC LNVGLIRKLS DFIDPQEGWK KLAVAIKKPS
GDDRYNQFHI RRFEALLQTG KSPTSELLFD WGTTNCTVGD LVDLLIQNEF
FAPASLLLPD AVPK

Red = MS-MS **Underline = PMF**

Comments: Individual ions scores > 20 indicate identity or extensive homology (p<0.05).

Score	Peptide	Modifications	Obs. m/z	Delta	MS-MS?	PMF?
47	-.GAMDPEFMNKPITPSTYVR.C	Phospho (ST)	745.34	-0.0003	☒	☐
(20)	-.GAMDPEFMNKPITPSTYVR.C	Phospho (ST)	1,117.51	0.0008	☒	☐
(22)	-.GAMDPEFMNKPITPSTYVR.C	Phospho (ST); Oxidation (M)	1,125.50	-0.0007	☒	☐
(24)	-.GAMDPEFMNKPITPSTYVR.C	Phospho (ST); Oxidation (M)	750.67	-0.0006	☒	☐
(26)	-.GAMDPEFMNKPITPSTYVR.C	Oxidation (M)	1,085.52	0.0037	☒	☐
(29)	-.GAMDPEFMNKPITPSTYVR.C	2 Oxidation (M)	729.35	0.0020	☒	☐
(32)	-.GAMDPEFMNKPITPSTYVR.C	Oxidation (M)	724.02	0.0009	☒	☐
(33)	-.GAMDPEFMNKPITPSTYVR.C		718.68	-0.0009	☒	☐
(33)	-.GAMDPEFMNKPITPSTYVR.C		1,077.52	0.0014	☒	☐
(33)	-.GAMDPEFMNKPITPSTYVR.C	2 Oxidation (M)	1,093.52	0.0066	☒	☐
(41)	-.GAMDPEFMNKPITPSTYVR.C	Phospho (ST); 2 Oxidation (M)	756.00	-0.0005	☒	☐
59	R.CLNVGLIR.K		472.77	-0.0006	☒	☐
(38)	R.CLNVGLIR.K		472.77	0.0001	☒	☐
(44)	R.CLNVGLIR.K		472.77	-0.0005	☒	☐
(49)	R.CLNVGLIR.K		472.77	0.0010	☒	☐
(50)	R.CLNVGLIR.K		472.77	0.0042	☒	☐
69	R.KLSDFIDPQEGWK.K		781.90	0.0014	☒	☐
22	R.KLSDFIDPQEGWKK.L		564.30	-0.0003	☒	☐
(21)	R.KLSDFIDPQEGWK.K		521.60	-0.0027	☒	☐
(67)	R.KLSDFIDPQEGWK.K		781.90	-0.0008	☒	☐
44	K.LSDFIDPQEGWK.K		717.85	-0.0012	☒	☐
34	K.LSDFIDPQEGWKK.L		781.90	0.0004	☒	☐
(25)	K.LSDFIDPQEGWKK.L		521.60	-0.0014	☒	☐
(38)	K.LSDFIDPQEGWK.K		717.85	0.0011	☒	☐
50	K.LAVAIKKPSGDDR.Y		457.26	0.0002	☒	☐
39	K.LAVAIK.K		307.72	-0.0006	☒	☐
(26)	K.LAVAIK.K		307.72	0.0005	☒	☐
40	K.KPSGDDR.Y		387.69	0.0002	☒	☐
30	K.KPSGDDRYNQFHIR.R		866.93	0.0014	☒	☐
(20)	K.KPSGDDRYNQFHIR.R		433.97	-0.0014	☒	☐
(20)	K.KPSGDDRYNQFHIR.R		578.29	-0.0011	☒	☐
(36)	K.KPSGDDR.Y		387.69	-0.0002	☒	☐
(36)	K.KPSGDDR.Y		387.69	0.0000	☒	☐
(36)	K.KPSGDDR.Y		387.69	0.0002	☒	☐
(40)	K.KPSGDDR.Y		387.69	0.0000	☒	☐

(b)

Acc. #	Protein ID	Pep. #	% Cov.	Score
9947	IRAK4 DEATH DOMAIN	59	62%	*645*

GAMDPEFMNK PITPSTYVRC LNVGLIRKLS DFIDPQEGWK KLAVAIKKPS
GDDRYNQFHI RRFEALLQTG KSPTSELLFD WGTTNCTVGD LVDLLIQNEF
FAPASLLLPD AVPK

Red = MS-MS **Underline = PMF**

Comments: Individual ions scores > 20 indicate identity or extensive homology (p<0.05).

Score	Peptide	Modifications	Obs. m/z	Delta	MS-MS?	PMF?
(36)	K.KPSGDDR.Y		387.69	0.0002	☒	☐
(40)	K.KPSGDDR.Y		387.69	0.0000	☒	☐
(40)	K.KPSGDDR.Y		387.69	0.0001	☒	☐
(40)	K.KPSGDDR.Y		387.69	0.0001	☒	☐
(40)	K.KPSGDDR.Y		387.69	0.0001	☒	☐
45	R.YNQFHIR.R		489.25	-0.0003	☒	☐
(20)	R.YNQFHIR.R		326.50	-0.0004	☒	☐
(23)	R.YNQFHIR.R		489.25	-0.0003	☒	☐
(28)	R.YNQFHIR.R		489.25	-0.0012	☒	☐
(28)	R.YNQFHIR.R		489.25	-0.0009	☒	☐
(28)	R.YNQFHIR.R		489.25	-0.0002	☒	☐
(30)	R.YNQFHIR.R		489.25	0.0000	☒	☐
(44)	R.YNQFHIR.R		489.25	-0.0011	☒	☐
54	R.RFEALLQTGK.S		581.83	-0.0012	☒	☐
(20)	R.RFEALLQTGK.S		581.83	-0.0001	☒	☐
(32)	R.RFEALLQTGK.S		388.22	0.0006	☒	☐
(37)	R.RFEALLQTGK.S		581.83	-0.0002	☒	☐
(42)	R.RFEALLQTGK.S		581.83	0.0001	☒	☐
(44)	R.RFEALLQTGK.S		581.83	0.0000	☒	☐
(46)	R.RFEALLQTGK.S		581.83	-0.0006	☒	☐
(46)	R.RFEALLQTGK.S		581.83	-0.0002	☒	☐
(54)	R.RFEALLQTGK.S		581.83	0.0006	☒	☐
55	R.FEALLQTGK.S		503.78	-0.0009	☒	☐
44	K.TANTLPSK.E		416.23	0.0002	☒	☐
27	K.EAITVQQK.Q		458.76	0.0000	☒	☐
47	K.SLEVSDTR.F		453.73	0.0001	☒	☐

(c)

9947	IRAK4 DEATH DOMAIN	88	62%	***645***

GAMDPEFMNK PITPSTYVRC LNVGLIRKLS DFIDPQEGWK KLAVAIKKPS
GDDRYNQFHI RRFEALLQTG KSPTSELLFD WGTTNCTVGD LVDLLIQNEF
FAPASLLLPD AVPK

Red = MS-MS **Underline = PMF**

Comments: Individual ions scores > 20 indicate identity or extensive homology (p<0.05).

Score	Peptide	Modifications	Obs. m/z	Delta	MS-MS?	PMF?
47	K.LAVAIK.K		307.72	-0.0001	☒	☐
(29)	K.LAVAIK.K		307.72	-0.0002	☒	☐
(33)	K.LAVAIK.K		307.72	0.0000	☒	☐
(43)	K.LAVAIK.K		307.72	0.0005	☒	☐
(45)	K.LAVAIK.K		307.72	0.0005	☒	☐
40	K.KPSGDDR.Y		387.69	0.0000	☒	☐
34	K.KPSGDDRYNQFHIR.R		866.93	0.0030	☒	☐
(25)	K.KPSGDDRYNQFHIR.R		578.29	0.0014	☒	☐
(37)	K.KPSGDDR.Y		387.69	0.0002	☒	☐
(39)	K.KPSGDDR.Y		387.69	0.0002	☒	☐
(40)	K.KPSGDDR.Y		387.69	0.0001	☒	☐
(40)	K.KPSGDDR.Y		387.69	0.0001	☒	☐
(40)	K.KPSGDDR.Y		387.69	0.0002	☒	☐
50	R.YNQFHIR.R		489.25	-0.0001	☒	☐
(25)	R.YNQFHIR.R		489.25	-0.0007	☒	☐
(29)	R.YNQFHIR.R		489.25	0.0005	☒	☐
(31)	R.YNQFHIR.R		489.25	-0.0003	☒	☐
(34)	R.YNQFHIR.R		489.25	-0.0003	☒	☐
(36)	R.YNQFHIR.R		489.25	0.0010	☒	☐
(38)	R.YNQFHIR.R		489.25	0.0001	☒	☐
(38)	R.YNQFHIR.R		489.25	0.0005	☒	☐
(41)	R.YNQFHIR.R		489.25	-0.0007	☒	☐
(41)	R.YNQFHIR.R		489.25	-0.0002	☒	☐
(41)	R.YNQFHIR.R		489.25	-0.0002	☒	☐
(45)	R.YNQFHIR.R		489.25	-0.0005	☒	☐

(d)

9947	IRAK4 DEATH DOMAIN	88	62%	***645***

GAMDPEFMNK PITPSTYVRC LNVGLIRKLS DFIDPQEGWK KLAVAIKKPS
GDDRYNQFHI RRFEALLQTG KSPTSELLFD WGTTNCTVGD LVDLLIQNEF
FAPASLLLPD AVPK

Red = MS-MS **Underline = PMF**

Comments: Individual ions scores > 20 indicate identity or extensive homology (p<0.05).

Score	Peptide	Modifications	Obs. m/z	Delta	MS-MS?	PMF?
(46)	R.YNQFHIR.R		489.25	-0.0005	☒	☐
(47)	R.YNQFHIR.R		489.25	-0.0010	☒	☐
67	R.RFEALLQTGK.S		581.83	-0.0010	☒	☐
(20)	R.RFEALLQTGK.S		388.22	0.0011	☒	☐
(31)	R.RFEALLQTGK.S		581.83	0.0010	☒	☐
(36)	R.RFEALLQTGK.S		388.22	-0.0005	☒	☐
(50)	R.RFEALLQTGK.S		581.83	-0.0003	☒	☐
(51)	R.RFEALLQTGK.S		581.83	0.0005	☒	☐
(55)	R.RFEALLQTGK.S		581.83	-0.0002	☒	☐
(55)	R.RFEALLQTGK.S		581.83	-0.0002	☒	☐
(55)	R.RFEALLQTGK.S		581.83	-0.0001	☒	☐
(55)	R.RFEALLQTGK.S		581.83	0.0003	☒	☐
(55)	R.RFEALLQTGK.S		581.83	0.0003	☒	☐
(55)	R.RFEALLQTGK.S		581.83	0.0003	☒	☐
(55)	R.RFEALLQTGK.S		581.83	0.0004	☒	☐
(56)	R.RFEALLQTGK.S		581.83	0.0004	☒	☐
(56)	R.RFEALLQTGK.S		581.83	0.0004	☒	☐
(61)	R.RFEALLQTGK.S		581.83	0.0003	☒	☐
(61)	R.RFEALLQTGK.S		581.83	0.0004	☒	☐
(65)	R.RFEALLQTGK.S		581.83	-0.0001	☒	☐
55	R.FEALLQTGK.S		503.78	-0.0019	☒	☐
(20)	R.FEALLQTGK.S		503.78	-0.0001	☒	☐
(27)	R.FEALLQTGK.S		503.78	0.0000	☒	☐
(42)	R.FEALLQTGK.S		503.78	-0.0006	☒	☐

Table 3: Peptides identified by MALDI-TOF MS after digestion of IRAK-4 DD using Trypsin.

9947	IRAK4 DEATH DOMAIN	19	62%	***427***

GAMDPEFMNK PITPSTYVRC LNVGLIRKLS DFIDPQEGWK KLAVAIKKPS
GDDRYNQFHI RRFEALLQTG KSPTSELLFD WGTTNCTVGD LVDLLIQNEF
FAPASLLLPD AVPK

Red = MS-MS **Underline = PMF**

Comments: Individual ions scores > 28 indicate identity or extensive homology ($p<0.05$).

Score	Peptide	Modifications	Obs. m/z	Delta	MS-MS?	PMF?
77	-.GAMDPEFMNKPITPSTYVR.C		2,154.14	0.1071	☒	☐
0	-.GAMDPEFMNKPITPSTYVR.C	Oxidation (M)	2,170.15	0.1183	☐	☒
0	-.GAMDPEFMNKPITPSTYVR.C	2 Oxidation (M)	2,186.16	0.1288	☐	☒
(48)	-.GAMDPEFMNKPITPSTYVR.C	Phospho (ST)	2,234.13	0.1295	☒	☐
(57)	-.GAMDPEFMNKPITPSTYVR.C	Oxidation (M); Phospho (ST)	2,250.12	0.1276	☒	☐
(74)	-.GAMDPEFMNKPITPSTYVR.C	2 Oxidation (M); Phospho (ST)	2,266.13	0.1421	☒	☐
0	R.CLNVGLIR.K		944.58	0.0449	☐	☒
92	R.KLSDFIDPQEGWKK.L		1,690.96	0.0765	☒	☐
73	R.KLSDFIDPQEGWK.K		1,562.86	0.0785	☒	☐
0	R.KLSDFIDPQEGWKK.L	Phospho (ST)	1,770.89	0.0462	☐	☒
0	K.LSDFIDPQEGWK.K		1,434.77	0.0769	☐	☒
0	K.LAVAIKKPSGDDRYNQFHIR.R		2,328.40	0.1441	☐	☒
0	K.LAVAIKKPSGDDRYNQFHIRR.F	Phospho (ST)	2,564.29	-0.0364	☐	☒
89	K.KPSGDDRYNQFHIR.R		1,732.96	0.1135	☒	☐
0	K.KPSGDDR.Y	Phospho (ST)	854.32	-0.0173	☐	☒
0	K.KPSGDDRYNQFHIRR.F		1,889.05	0.0922	☐	☒
44	R.YNQFHIR.R		977.54	0.0472	☒	☐
0	R.YNQFHIRR.F		1,133.64	0.0386	☐	☒
52	R.RFEALLQTGK.S		1,162.72	0.0650	☒	☐

Table 4: Peptides identified by MALDI-TOF MS after digestion of IRAK-4 DD using Chymotrypsin.

9947	IRAK4 DEATH DOMAIN	20	80%	***357***

GAMDPEFMNK PITPSTYVRC LNVGLIRKLS DFIDPQEGWK KLAVAIKKPS
GDDRYNQFHI RRFEALLQTG KSPTSELLFD WGTTNCTVGD LVDLLIQNEF
FAPASLLLPD AVPK

Red = MS-MS **Underline = PMF**

Comments: Individual ions scores > 32 indicate identity or extensive homology ($p<0.05$).

Score	Peptide	Modifications	Obs. m/z	Delta	MS-MS?	PMF?
34	-.GAMDPEFMNKPITPSTY.V	2 Oxidation (M); Phospho (ST)	2,010.89	0.0626	☒	☐
0	-.GAMDPEF.M		766.38	0.0711	☐	☒
0	-.GAMDPEFMNKPITPSTY.V		1,898.92	0.0535	☐	☒
0	-.GAMDPEFMNKPITPSTY.V	Oxidation (M)	1,914.91	0.0525	☐	☒
0	-.GAMDPEFMNKPITPSTY.V	2 Oxidation (M)	1,930.91	0.0573	☐	☒
79	L.NVGLIRKLSDFIDPQEGW.K		2,087.17	0.0762	☒	☐
0	L.NVGLIRKL.S		912.62	0.0219	☐	☒
69	L.IRKLSDFIDPQEGW.K		1,703.92	0.0454	☒	☐
0	L.IRKLSDFIDPQEGWKKL.A		2,073.20	0.0546	☐	☒
0	L.SDFIDPQEGWKKL.A		1,562.82	0.0359	☐	☒
0	W.KKLAVAIKKPSGDDRY.N		1,789.07	0.0377	☐	☒
0	W.KKLAVAIKKPSGDDRYNQF.H		2,178.27	0.0699	☐	☒
78	L.AVAIKKPSGDDRYNQF.H		1,808.98	0.0558	☒	☐
55	L.AVAIKKPSGDDRY.N		1,419.80	0.0398	☒	☐
0	Y.NQFHIRRF.E		1,117.63	0.0318	☐	☒
0	F.HIRRF.E		728.43	-0.0031	☐	☒
0	F.HIRRFEAL.L		1,041.62	0.0269	☐	☒
0	F.HIRRFEALL.Q		1,154.72	0.0354	☐	☒
0	L.QTGKSPTSELLFDW.G	Phospho (ST)	1,688.90	0.1433	☐	☒
42	F.FAPASLLLPDAVPK.-		1,438.87	0.0371	☒	☐

Table 5: Peptides identified by LC / MS - MS after digestion of IRAK-4 DD using Asp-N.

9947	IRAK4 DEATH DOMAIN	11	82%	***280***

GAMDPEFMNK PITPSTYVRC LNVGLIRKLS DFIDPQEGWK KLAVAIKKPS
GDDRYNQFHI RRFEALLQTG KSPTSELLFD WGTTNCTVGD LVDLLIQNEF
FAPASLLLPD AVPK

Red = MS-MS **Underline = PMF**

Comments: Individual ions scores > 19 indicate identity or extensive homology (p<0.05).

Score	Peptide	Modifications	Obs. m/z	Delta	MS-MS?	PMF?
19	M.DPEFMNKPITPSTYVRCLNVGLIRKLS.D	Oxidation (M)	791.92	0.0028	☒	☐
25	S.DFIDPQEGWKKLAVAIKKPSG.D		776.43	0.0013	☒	☐
31	I.DPQEGWKKLAVAIKKPSG.D		651.37	0.0031	☒	☐
29	I.DPQEGWKKLAVAIKKPSGD.D		689.71	-0.0010	☒	☐
(28)	I.DPQEGWKKLAVAIKKPSG.D		976.56	0.0066	☒	☐
23	Q.EGWKKLAVAIKKPSG.D		403.75	0.0006	☒	☐
31	G.DDRYNQFHIRRF.E		417.46	-0.0004	☒	☐
(24)	G.DDRYNQFHIRRF.E		417.46	0.0006	☒	☐
67	F.EALLQTGKSPTS.E		616.33	-0.0008	☒	☐
22	V.DLLIQNEFFAPASLLLPDAVPK.-		804.45	0.0013	☒	☐
33	N.EFFAPASLLLPDAVPK.-		857.98	0.0015	☒	☐

Table 6: Peptides identified by MALDI-TOF MS after digestion of IRAK-4 DD using Asp-N.

9947	IRAK4 DEATH DOMAIN	5	63%	***249***

GAMDPEFMNK PITPSTYVRC LNVGLIRKLS DFIDPQEGWK KLAVAIKKPS
GDDRYNQFHI RRFEALLQTG KSPTSELLFD WGTTNCTVGD LVDLLIQNEF
FAPASLLLPD AVPK

Red = MS-MS **Underline = PMF**

Comments: Individual ions scores > 23 indicate identity or extensive homology (p<0.05).

Score	Peptide	Modifications	Obs. m/z	Delta	MS-MS?	PMF?
0	M.DPEFMNKPITPSTYVRCLNVGLIRKLS.D	Oxidation (M)	3,164.86	0.1989	☐	☒
134	S.DFIDPQEGWKKLAVAIKKPSG.D		2,327.43	0.1534	☒	☐
115	I.DPQEGWKKLAVAIKKPSG.D		1,952.22	0.1280	☒	☐
0	G.DDRYNQFHIRRF.E		1,666.92	0.1008	☐	☒
0	D.DRYNQFHIRRFEALLQTGKSPTS.E	Phospho (ST)	2,844.30	-0.0975	☐	☒

Table 7: MS/MS Fragmentation of GAMDPEFMNKPITPSTYVR

(a) Peptide fragment spectrum of b- and y- ion series corresponding to the cleavage of the amide bond at the N-terminus and C-terminus respectively. (b) Table of the corresponding observed fragmentation masses showing matching peptide hits in Bold Red: 45/272 fragment ions using 125 most intense peaks used to give an ion score of 20 in the MASCOT database. (c) Zoomed area of peptide fragment spectrum from 1600 to 1750 m/z showing the ion b*(15) corresponding to the loss of 98 Da at Ser8. (d) Zoomed area of peptide fragment spectrum from 300 to 400 m/z showing the ion $y^{++}(5)$ corresponding to the loss of 98 Da at Ser8.

(a)

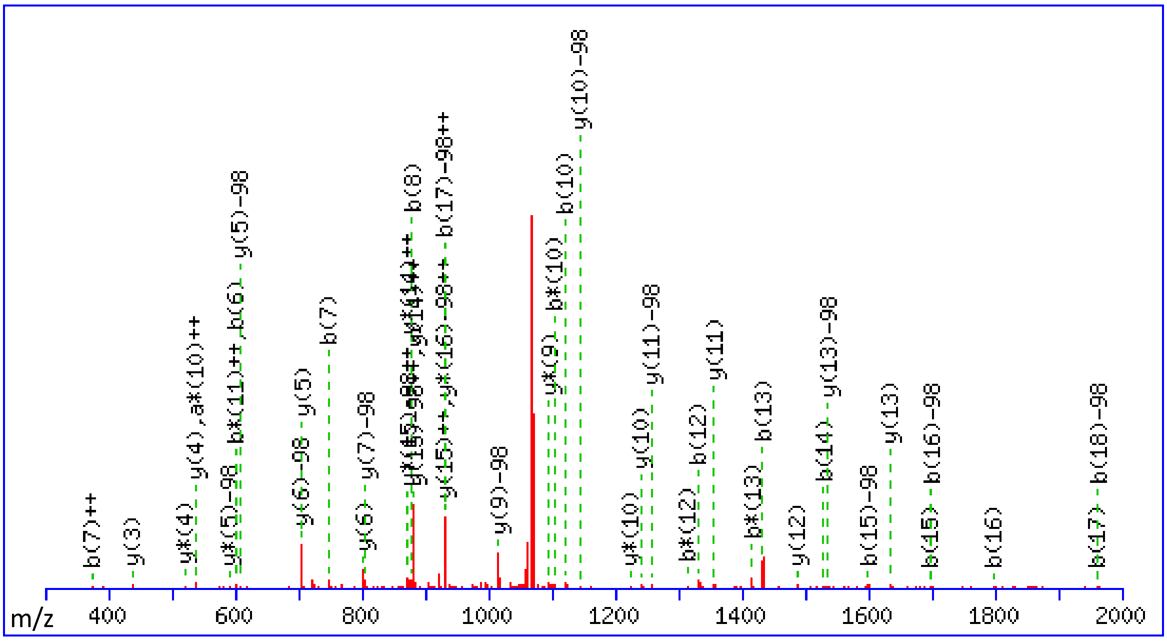

(b)

#	b	b^{++}	b*	b^{*++}	Seq.	y	y^{++}	y*	y^{*++}	#
1	58.0287	29.5180			G					19
2	129.0659	65.0366			A	2176.9807	1088.9940	2159.9541	1080.4807	18
3	**260.1063**	130.5568			M	2105.9436	**1053.4754**	2088.9170	1044.9621	17
4	**375.1333**	188.0703			D	1974.9031	**987.9552**	**1957.8765**	979.4419	16
5	**472.1860**	236.5967			P	**1859.8761**	**930.4417**	1842.8496	921.9284	15
6	**601.2286**	301.1180			E	1762.8234	**881.9153**	1745.7968	873.4021	14
7	748.2971	**374.6522**			F	1633.7808	**817.3940**	1616.7542	808.8808	13
8	**879.3375**	440.1724			M	1486.7124	743.8598	1469.6858	735.3465	12
9	**993.3805**	497.1939	976.3539	488.6806	N	1355.6719	**678.3396**	1338.6453	669.8263	11
10	**1121.4754**	561.2414	1104.4489	552.7281	K	1241.6290	**621.3181**	1224.6024	**612.8048**	10
11	1218.5282	609.7677	1201.5016	**601.2545**	P	**1113.5340**	**557.2706**	1096.5074	548.7574	9
12	**1331.6123**	666.3098	1314.5857	657.7965	I	**1016.4812**	**508.7443**	999.4547	500.2310	8
13	**1432.6599**	**716.8336**	**1415.6334**	708.3203	T	**903.3972**	**452.2022**	886.3706	443.6889	7
14	1529.7127	765.3600	1512.6861	756.8467	P	**802.3495**	**401.6784**	785.3229	393.1651	6
15	1696.7111	848.8592	**1679.6845**	840.3459	S	705.2967	**353.1520**	688.2702	344.6387	5
16	**1797.7587**	899.3830	1780.7322	890.8697	T	**538.2984**	269.6528	521.2718	261.1396	4
17	1960.8221	980.9147	1943.7955	972.4014	Y	**437.2507**	**219.1290**	420.2241	210.6157	3
18	2059.8905	1030.4489	2042.8639	1021.9356	V	**274.1874**	137.5973	257.1608	129.0840	2
19					R	175.1190	88.0631	158.0924	79.5498	1

(c)

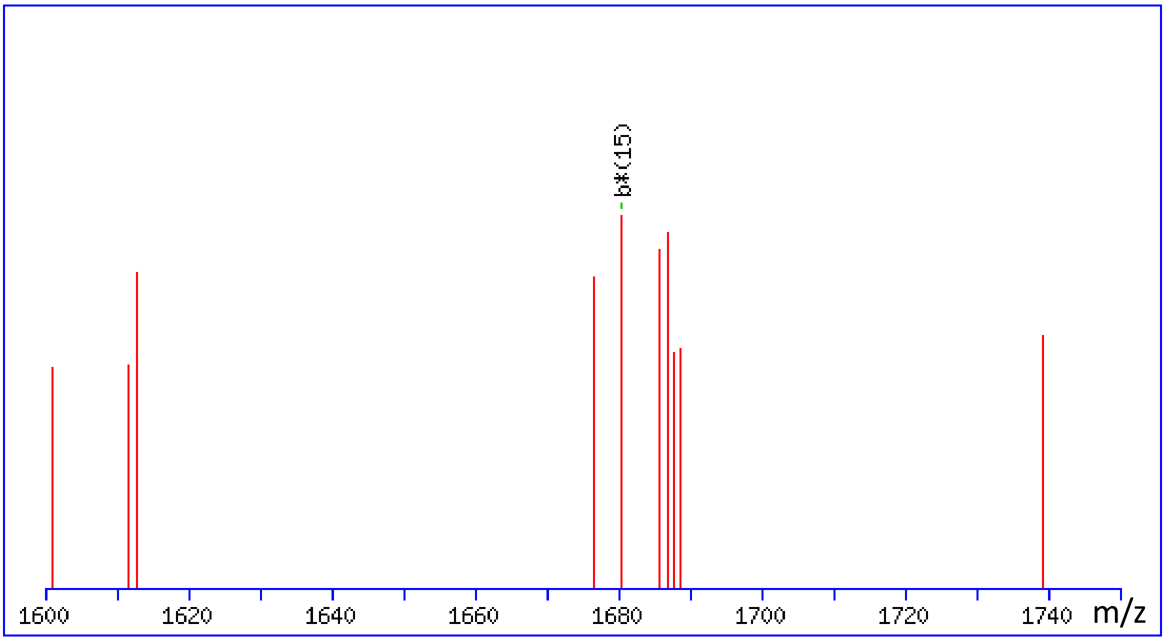
b*(15)
1600
1620
1640
1660
1680
1700
1720
1740
m/z

(d)

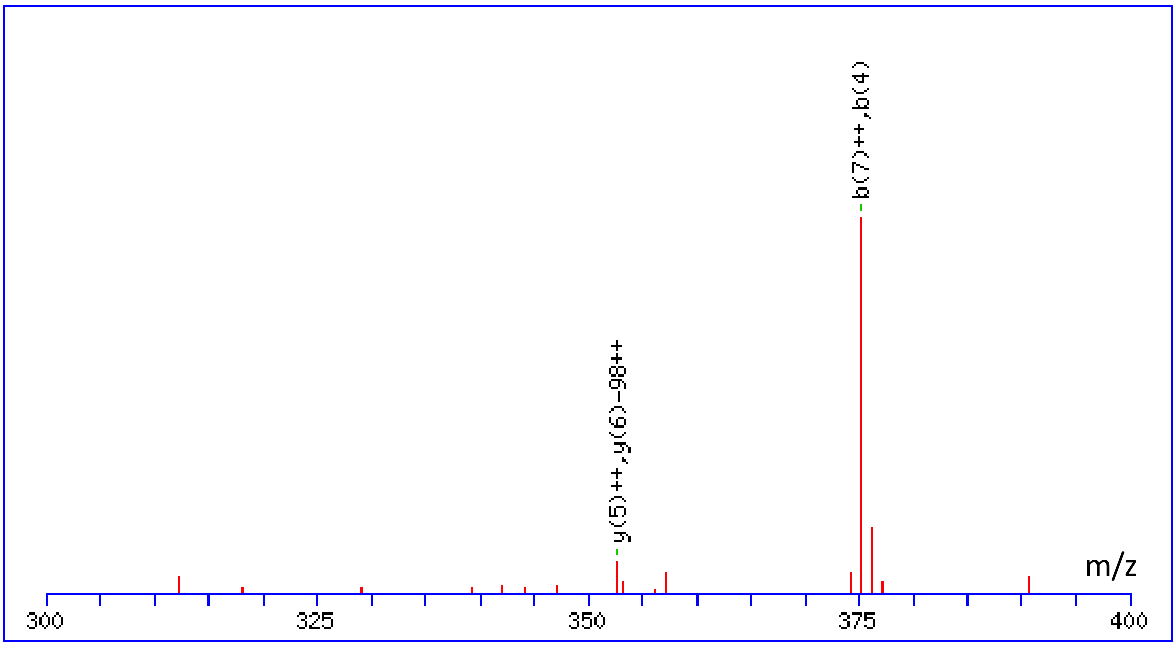
b(7)++,b(4)
y(5)++,y(6)-98++
m/z
300
325
350
375
400

Table 8: MS/MS Fragmentation of GAMDPEFMNKPITPSTYVR

(a) Peptide fragment spectrum of b- and y- ion series corresponding to the cleavage of the amide bond at the N-terminus and C-terminus respectively. (b) Table of the corresponding observed fragmentation masses showing matching peptide hits in 56/272 fragment ions using 129 most intense peaks used to give an ion score of 48 in the MASCOT database. (c) Zoomed area of peptide fragment spectrum from 1550 to 1650 m/z showing the ion b(15) corresponding to the loss of 98 Da at Ser8. (d) Zoomed area of peptide fragment spectrum from 550 to 650 m/z showing the ions y(5) and y*(5) corresponding to the loss of 98 Da at Ser8.

(a)

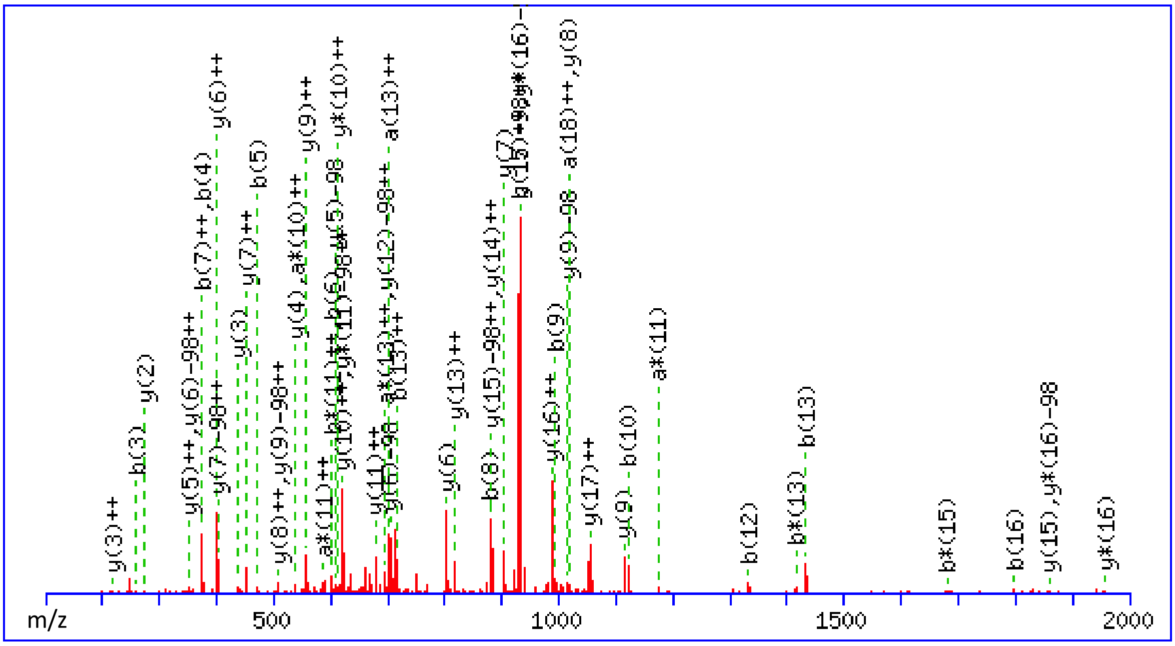

(b)

#	b	b^{++}	b*	b^{*++}	Seq.	y	y^{++}	y*	y^{*++}	#
1	58.0287	29.5180			G					19
2	129.0659	65.0366			A	2079.0038	1040.0055	2061.9772	1031.4923	18
3	260.1063	130.5568			M	2007.9667	1004.4870	1990.9401	995.9737	17
4	375.1333	188.0703			D	1876.9262	938.9667	1859.8996	**930.4535**	16
5	472.1860	236.5967			P	1761.8992	**881.4533**	1744.8727	**872.9400**	15
6	**601.2286**	301.1180			E	1664.8465	832.9269	1647.8199	824.4136	14
7	**748.2971**	**374.6522**			F	**1535.8039**	768.4056	1518.7773	759.8923	13
8	**879.3375**	440.1724			M	1388.7355	694.8714	1371.7089	686.3581	12
9	993.3805	497.1939	976.3539	488.6806	N	**1257.6950**	629.3511	1240.6684	620.8379	11
10	**1121.4754**	561.2414	**1104.4489**	552.7281	K	**1143.6521**	572.3297	1126.6255	563.8164	10
11	1218.5282	609.7677	1201.5016	**601.2545**	P	**1015.5571**	508.2822	998.5306	499.7689	9
12	**1331.6123**	666.3098	**1314.5857**	657.7965	I	918.5043	459.7558	901.4778	451.2425	8
13	**1432.6599**	716.8336	**1415.6334**	708.3203	T	**805.4203**	403.2138	788.3937	394.7005	7
14	**1529.7127**	765.3600	1512.6861	756.8467	P	**704.3726**	352.6899	687.3460	344.1767	6
15	**1598.7342**	799.8707	1581.7076	791.3574	S	**607.3198**	304.1636	**590.2933**	295.6503	5
16	**1699.7818**	850.3946	1682.7553	841.8813	T	**538.2984**	269.6528	**521.2718**	261.1396	4
17	1862.8452	**931.9262**	1845.8186	923.4129	Y	**437.2507**	219.1290	420.2241	210.6157	3
18	**1961.9136**	981.4604	1944.8870	972.9472	V	274.1874	137.5973	257.1608	129.0840	2
19					R	175.1190	88.0631	158.0924	79.5498	1

(c)

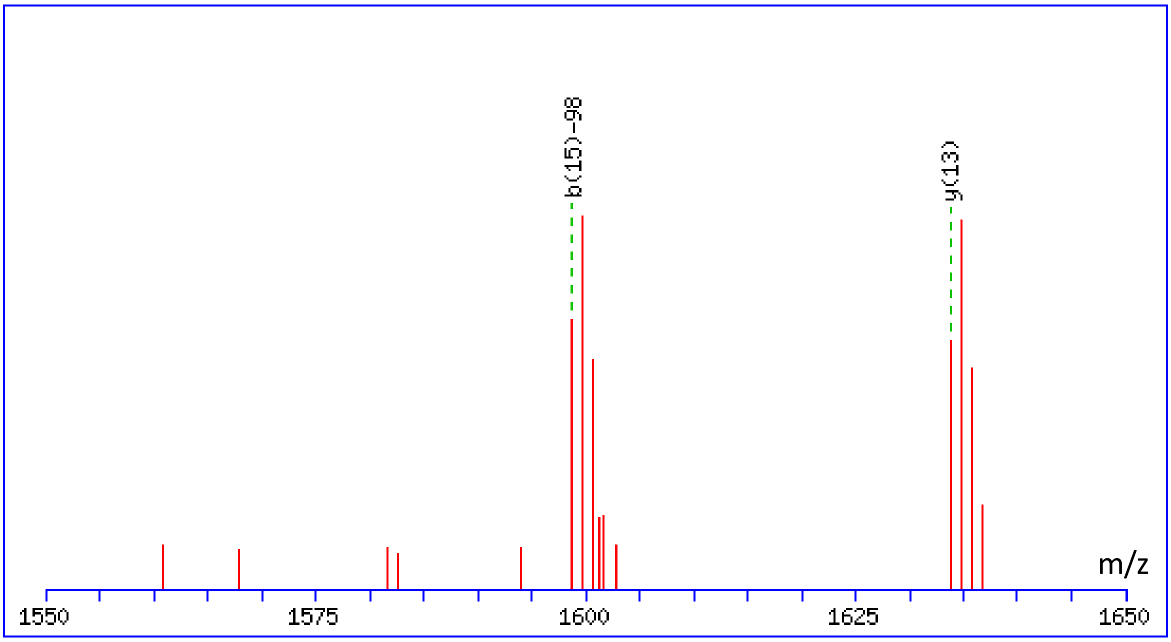

(d)

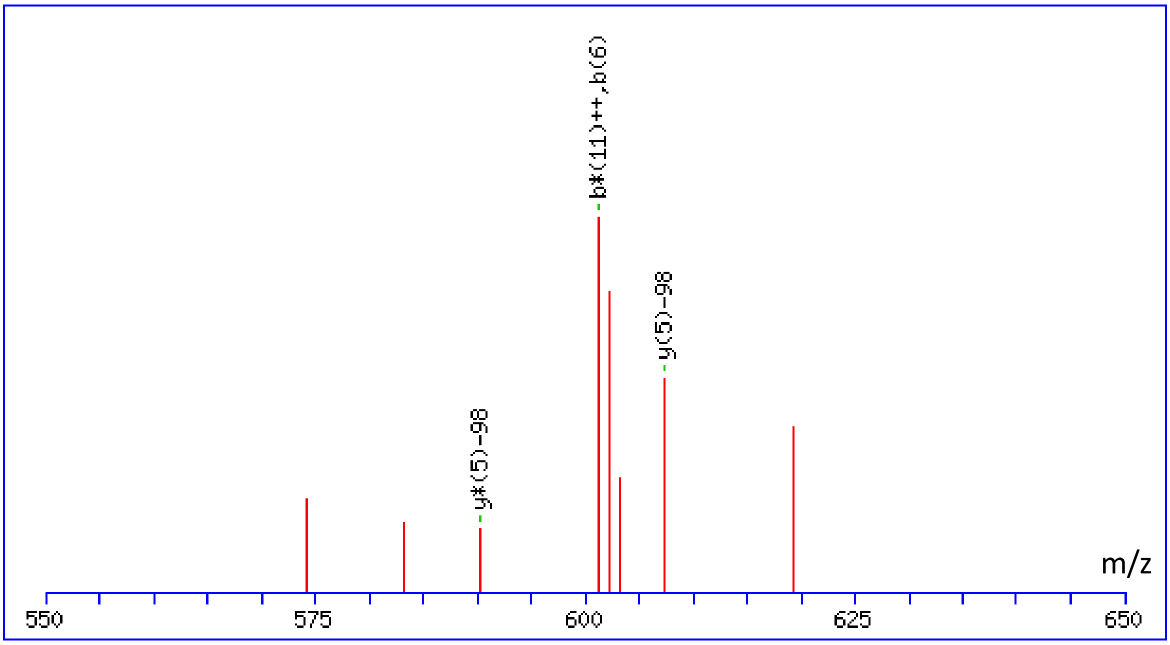

Supplementary information - Model validation.

After regularization in COOT, sidechain packing was optimised and total energy compared with the unphosphorylated IRAK4 structure (pdb id: 3mop, chain G) using the FoldX atomic forcefield. (FoldX version 4.0 running under Win10)[1]. Sidechain clashes were checked with 'findclash' function in UCSF-Chimera[2].

Neglecting the new stabilizing interactions of the phospho-serine residue, the helical refolding of the N-terminal 16 residues was calculated by FoldX to be comparable in energy to the crystal structure. Compared with the equivalent optimised crystal structure (minimised with the FoldX 'Repair' protocol), the refolded IRAK4 had an overall ΔΔG stabilisation of -0.2 kcal/mol (neglecting any phospho-serine contributions). This favourable change in estimated stability on helical refolding is for the IRAK4 in isolation, but no unfavourable contacts are generated when packed with other IRAK4 and IRAK2 chains to form the myddosome assembly.

The geometry and sidechain packing of the refolded IRAK4 were validated with WHATCHECK10.1 (http://swift.cmbi.ru.nl/gv/whatcheck/). Ramachandran analysis (with COOT) showed no new outliers were generated by the helix refolding: the IRAK4 crystal structure had 98 residues in preferred or allowed regions, and 7 outliers. None of the outliers is in the N-terminal region and refolding of the N-terminal region does not introduce any new Ramachandran outliers.

References

1. Kiel, C., Serrano, L. & Herrmann, C. A detailed thermodynamic analysis of ras/effector complex interfaces. *J. Mol. Biol.* **340**, 1039-1058 (2004).

2. Yang, Z. *et al.* UCSF Chimera, MODELLER, and IMP: an integrated modeling system. *J. Struct. Biol.* **179**, 269-278 (2012).
